# Frequency of common polymorphisms in ***Caveolin 1*** (***CAV1*** ) gene in adults with high serum triglycerides from Colombian Caribbean Coast

**DOI:** 10.25100/cm.v48i4.2625

**Published:** 2017-12-30

**Authors:** Gustavo Jose Mora-Garcia, Maria Stephany Ruiz-Diaz, Doris Esther Gomez-Camargo, Claudio Jaime Gomez-Alegria

**Affiliations:** 1 Doctorado en Medicina Tropical, Facultad de Medicina, Universidad de Cartagena,Cartagena de Indias, Colombia.; 2 Grupo de Investigación UNIMOL . Departamento de Farmacia, Facultad de Ciencias, Universidad Nacional de Colombia. Bogotá, Colombia.

**Keywords:** Hypertriglyceridemia, Caveolin 1, Single Nucleotide Polymorphism, Caribbean Region, Colombia, Metabolic diseases, Hipertrigliceridemia, Caveolina 1, Polimorfismos de Nucleótido Simple, Región Caribe, Colombia

## Abstract

**Background:**

Caveolin 1 gene (*CAV1*) has been associated with insulin resistance, metabolic syndrome and hypertension in humans. Also, it has been related to high serum triglycerides in rodents, however there is little evidence of this relation in humans.

**Aim:**

To describe frequencies of common variations in *CAV1* in adults with high serum triglycerides.

**Methods:**

A case-control study was carried out with adults from Colombian Caribbean Coast. A whole blood sample was employed to measure serum concentrations of triglycerides, glucose, total cholesterol and HDLc. Six common Single Nucleotide Polymorphism (SNP) in *CAV1* were genotyped (rs926198, rs3779512, rs10270569, rs11773845, rs7804372 and rs1049337). Allelic and genotypic frequencies were determined by direct count and Hardy-Weinberg Equilibrium (HWE) was assessed. Case and control groups were compared with null-hypothesis tests.

**Results:**

A total of 220 cases and 220 controls were included. For rs3779512 an excess in homozygotes frequency was found within case group (40.4% (GG), 41.3% (GT) and 18.1% (TT); F_is_=0.13, p=0.03). Another homozygotes excess among case group was found in rs7804372 (59.5% (TT), 32.3% (TA) and 8.2% (AA); F_is_= 0.12, *p*= 0.04). In rs1049337, cases also showed an excess in homozygotes frequency (52.7% (CC), 35.0% (CT) and 12.3% (TT); F_is_= 0.16, *p*= 0.01). Finally, for rs1049337 there were differences in genotype distribution between case and control groups (*p* <0.05).

**Conclusion:**

An increased frequency of homozygote genotypes was found in subjects with high serum triglycerides. These findings suggest that minor alleles for SNPs rs3779512, rs7804372 and rs1049337 might be associated to higher risk of hypertriglyceridemia.

## Resumen

**Introducción:**

En humanos, el gen Caveolina 1 (*CAV1*) ha sido asociado con resistencia a la insulina, síndrome metabólico e hipertensión. Además, ha sido relacionado con hipertrigliceridemia en roedores, sin embargo existe poca evidencia de esta relación en humanos.

**Objetivo:**

Describir la frecuencia de variaciones comunes del gen *CAV1* en adultos con hipertrigliceridemia.

**Métodos:**

Se realizó un estudio de casos y controles con adultos del Caribe Colombiano. Fue usada una muestra de sangre venosa periférica para medir las concentraciones séricas de triglicéridos, glucosa, colesterol total y colesterol HDL. Fueron genotipificados seis Polimorfismos de Nucleótido Simple (SNP) en *CAV1* (rs926198, rs3779512, rs10270569, rs11773845, rs7804372 y rs1049337). Las frecuencias alélicas y genotípicas se determinaron por conteo directo y se evaluó el equilibrio de Hardy-Weinberg. Los grupos de casos y controles se compararon con pruebas de hipótesis nula.

**Resultados:**

Se incluyeron un total de 220 casos y 220 controles. Para rs3779512 se encontró un exceso de homocigotos en el grupo de casos (40.4% (GG), 41.3% (GT) y 18.1% (TT); F_is_= 0.13, *p*= 0.03). Fue encontrado otro exceso de homocigotos en el grupo de casos al analizar el rs7804372 (59.5% (TT), 32.3% (TA) y 8.2% (AA); F_is_= 0.12, *p*= 0.04). En rs1049337, los casos también tuvieron un exceso en la frecuencia de homocigotos (52.7% (CC), 35.0% (CT) y 12.3% (TT); F_is_= 0.16, *p*= 0.01). Finalmente, hubo diferencias en la distribución genotípica del rs1049337 entre los grupos de casos y controles (*p* <0.05).

**Conclusiones:**

Se encontró una elevada frecuencia de homocigotos en los sujetos con hipertrigliceridemia. Estos hallazgos sugieren que los alelos menores de los SNPs rs3779512, rs7804372 y rs1049337 podrían estar asociados con trigliceridemia elevada.

## Introduction

*Caveolin 1* (*CAV1*) is a 36.4 Kb gene located in chromosome 7 (7q31.1), with three exonic segments separated by two introns, which codifies for a 22 KDa membrane protein ^1^ ^-^ ^3^. These three exons account for 30, 165 and 342 bp, while introns represent more than 95% of total *CAV1* length and contain more than 500 Single Nucleotide Polymorphisms (SNP) ^1^ ^,^ ^4^.

CAVEOLIN-1 protein is indispensable for caveolae formation in plasma membrane of many cellular types, however adipose tissue expresses the highest amount of this protein in humans, rodents and other animals, suggesting that CAVEOLIN-1 and caveolae are involved in energy homeostasis ^5^ ^,^ ^6^. Moreover, *in vitro* studies demonstrated that CAVEOLIN-1 expression is significantly increased during adipogenesis, also it has been saw that the presence of this protein is necessary for appropriated preadipocytes proliferation and differentiation ^7^ ^,^ ^8^.

In adipocytes, CAVEOLIN-1 has been directly linked to insulin receptor as an enhancing factor for intrinsic phosphorylation ^9^ ^-^ ^11^, and it has been associated with stability of the Glucose Transpoter 4 (GLUT 4) ^7^ ^,^ ^12^. Additionally, CAVEOLIN-1 and caveolae are closely implicated in triglycerides metabolism through their association with lipid droplets conformation, and also caveolar domains in cell membrane are suspected to be sites for fatty acids entry and for triglycerides synthesis ^13^ ^-^ ^15^.

Animal models with rodents have demonstrated that caveolin-1 is crucial for triglycerides metabolism in adipose tissue since it seems to be involved in lipid droplets distribution and lipid storage dynamics ^16^. Therefore it has been found that *CAV1* knock-out individuals under an exposition to high fat diet are lean and more likely to develop hypertrygliceridemia as well as insulin resistance ^16^ ^,^ ^17^. 

In humans, common variations in *CAV1* gene have been associated to metabolic disorders such as insulin resistance, high blood pressure and metabolic syndrome ^18^ ^-^ ^21^. Initially two exonic variations in *CAV1* were associated with high blood pressure and metabolic syndrome in a population from Southern Spain ^18^. After that, an intronic SNP (rs926198) was associated with insulin resistance, and partially related to high blood pressure in a translational study that employed a human cohort with European ancestry ^20^. Recently, the same intronic polymorphism was related to metabolic syndrome, type 2 diabetes and low HDL cholesterol in a human cohort with European ancestry, and these findings were replicated in an admixed Hispanic group, which represents the first evidence involving *CAV1* with lipid alterations in humans ^19^. 

In spite of growing evidence linking *CAV1* with lipid homeostasis, there are still little findings supporting an association with triglycerides metabolism in human groups. Triglycerides (or triacylglycerol), the most relevant molecule for energy storage, are mainly accumulated in adipose tissue, but also are constitutive components of blood plasma and serum ^22^. High serum triglycerides (or hypertriglyceridemia) is a prevalent alteration among adult population that is generally associated with a high-energy unbalanced environment ^23^. In developing countries, dyslipidemias and particularly high serum triglycerides are public health issues, which contribute to cardiovascular mortality among more vulnerable communities ^24^. 

Together with most of Latin American countries, Colombia has registered a permanent elevation in frequencies of hypertriglyceridemia with notable intra-national variations in a range of prevalence that has been found as low as 19.3% and as high as 43.9% ^25^ ^,^ ^26^. In Cartagena de Indias, on the Colombian Caribbean Coast, high serum triglycerides were found in 38.2% of women ^27^, and between 67.7% and 77.8% of the general population ^28^, which represents one of the highest national frequencies. 

Considering that Colombian Caribbean populations would require novel strategies to reduce the impact of high serum triglycerides on public wellness, it has been proposed that genetic factors might be a source of valuable data to design preventive interventions or therapeutic approaches. In this way, this study was aimed to describe frequencies of six SNPs in *CAV1* in adults with high serum triglycerides from the Colombian Caribbean Coast.

## Materials and Methods

A cross-sectional, case-control study was carried out in urban zone of Cartagena de Indias, an admixed population with a predominant European ancestry (60%) as a consequence of Spaniards colonization between XVI and XIX centuries ^29^. 

Non-sibling individuals from both genders, aging 18-80 years old were included. Subjects with primary endocrine alterations or previous diagnosis of genetic disease, eating behavior disorders, recent hospitalization, cancer, personal antecedents of surgical treatment for obesity, pregnant women, and breast-feeding mothers were excluded. All subjects were asked to participate, and their informed consent was required, following Universidad de Cartagena Ethics Committee recommendations. 

Subjects were selected through a convenience sampling in work places and household clusters. For this procedure seven sampling points were included, which represent a socio-economic mixed population that is currently employed in diverse labors (*e.g.,* management, civil construction, industrial production, security services, and teaching/learning). Unemployed populations were included through a random cluster sampling that was executed in five socio-demographic diverse areas.

Classification of cases and control groups was based on Joint Interim Statement (JIS) criteria for high serum triglycerides ^30^. Therefore, cases were those subjects with serum triglycerides ≥150 mg/dL or clinical antecedents of high serum triglycerides, while subjects with serum concentrations below 150 mg/dL and without previous diagnosis of dyslipidemia were selected as controls. 

Fasting blood sample were collected for measuring of serum glucose, cholesterol, triglycerides and HDL cholesterol. Colorimetric assays to determine serum concentrations were performed in UNIMOL laboratory at the University of Cartagena following standard protocols. Serum biochemical parameters were assessed in order to bring a wide description of metabolic status. Among of these variables only serum triglycerides was employed as selection criteria for cases or controls. 

Socio-demographic variables were registered employing a validated survey previously applied to Caribbean urban communities ^27^ ^,^ ^28^. Cases and controls groups were paired by sex and age according to this data.

For genetic analysis, 3 mL of whole blood were collected in sterile tubes containing EDTA as anticoagulant; these samples were refrigerated and transported to UNIMOL laboratory at the University of Cartagena for further procedures. Genomic DNA was isolated through the employment of a commercial kit (Promega Corp., Madison, USA) following principles published by others ^31^, and then quantified through fluorometric method with Qubit 2.0 (Thermo Fisher Scientific Inc., Waltham, USA) using proper reagents.

For genotyping assay, prior to molecular procedures, common variants in *CAV1* were selected employing data from HapMap project and *Haploview 4.2 software* (*Broad Institute*, Cambridge, USA) ^4^ ^,^ ^32^. Genomic data from CEU population (Utah Residents with Northern and Western European Ancestry) was used as reference to select SNPs with Minor Allele Frequency (MAF) ≥0.25, and correlation coefficient (r^2)^ ≥0.8. According to these parameters, six polymorphisms were selected for further molecular analysis (Table 1). 

**Table 1:** Single Nucleotide Polymorphisms (SNP) selected in Caveolin 1 (CAV1) gene employing data from HapMap project. Minor Allele Frequency (MAF) ≥0.25 and r2 ≥0.8 were used as tagging criteria.

SNP	MAF	Function	Reference Variation
rs926198	0.332	Intron	C/T
rs3779512	0.407	Intron	G/T
rs10270569	0.274	Intron	C/T
rs11773845	0.420	Intron	A/C
rs7804372	0.270	Intron	A/T
rs1049337	0.250	3'-UTR	C/T

UTR: Untranslated Region

Selected SNPs were genotyped through quantitative Polymerase Chain Reaction (qPCR), using specific *TaqMan* probes (Thermo Fisher Scientific Inc., Waltham, USA). Allelic discrimination was automatically performed with end-point fluorescent data, which was analyzed through *StepOne Real-Time PCR Software* (Thermo Fisher Scientific Inc., Waltham, USA). 

Sociodemographic data, anthropometric measures and serum concentrations were described employing main tendency and frequency values. When appropriate, mean values were compared through Student's t test, while frequencies were compared with X^2^ or Fisher's exact tests. Allelic and genotypic frequencies were determined by direct count, linkage disequilibrium was estimated employing Arlequin 3.5 ^33^, and Hardy-Weinberg Equilibrium (HWE) was assessed through F_is_ values with Genetix 4.05 software. Differences in genotype distributions among cases and controls groups were assessed through null-hypothesis tests, as it has been described elsewhere ^34^.

## Results

A total of 440 subjects were included in the study (220 cases and 220 controls). Mean values for age, Body Mass Index (BMI), anthropometric and serum biochemical variables were represented in the Table 2. There were not statistically significant differences in sex distribution, age and anthropometric variables between both groups. 

**Table 2:** Socio-demographic, anthropometric and metabolic variables. Comparisons between groups were performed with X^2^ and t-student test, when appropriate.

Variables	Cases		Controls		*p*-value
Sex (males)	61.3%	54.5-67.7	66.3%	59.6-72.5	0.321
Age (years old)*	43.1	±12.0	42.1	±14.3	0.418
Anthropometric Parameters*					
Body Mass Index (kg/m^2)^	26.9	±4.0	26.3	±4.4	0.139
Waist Circumference (cm)	94.3	±9.5	92.4	±10.9	0.063
Hip Perimeter (cm)	101.9	±8.5	101.9	±9.1	0.941
Blood Pressure (mmHg)*					
Systolic	114.5	±16.8	111.9	±15.1	0.082
Diastolic	76.5	±11.3	75.0	±10.1	0.139
Serum Concentrations (mg/dL)*					
Triglycerides	210.4	±63.4	143.2	±27.2	<0.001
Glucose	100.7	±33.1	87.4	±25.5	<0.001
Cholesterol	200.9	±68.3	178.1	±37.2	<0.001
HDLc	47.4	±16.3	42.9	±10.9	<0.001

Sex was represented as proportion. 95% confidence interval

* Mean values ± standard deviation

For polymorphism rs926198, minor allele (C) was found in 27.9% of cases and in 30.9% of control subjects (*p*= 0.34). In the group of cases, genotypes TT, TC and CC were found in 52.7%, 38.6% and 8.6% of subjects, respectively; while in controls these genotypes were found in 50.0%, 38.2% and 11.8%, respectively. Hardy-Weinberg equilibrium (HWE) was found in both groups with F_is_ values in 0.04 (*p*= 0.31) and 0.10 (*p*= 0.07) for cases and controls, respectively (Table 3).

**Table 3:** Genotype distributions for Single Nucleotide Polymorphisms in Caveolin 1 (*CAV1*) gene in subjects with high serum triglycerides (cases) and controls. A recessive model was applied in order to compare differences between cases and controls.

Genotype	Cases	Controls	*p*-value
rs926198	n(%)	n(%)	
TT	116 (52.7)	110 (50.0)	
TC	85 (38.6)	84 (38.2)	
CC	19 (8.6)	26 (11.8)	0.53
TT+TC	201 (91.4)	194 (88.2)	
CC	19 (8.6)	26 (11.8)	0.34
rs3779512			
GG	89 (40.4)	82 (37.3)	
GT	91 (41.4)	101 (45.9)	
TT	40 (18.2)	37 (16.8)	0.62
GG+GT	180 (81.8)	183 (83.2)	
TT	40 (18.2)	37 (16.8)	0.70
rs10270569			
CC	149 (67.7)	141 (64.1)	
CT	61 (27.7)	75 (34.1)	
TT	10 (4.6)	4 (1.8)	0.12
CC+CT	210 (95.4)	216 (98.2)	
TT	10 (4.6)	4 (1.8)	0.17
rs11773845			
AA	88 (40.0)	71 (32.3)	
AC	92 (41.8)	96 (43.6)	
CC	40 (18.2)	53 (24.1)	0.15
AA+AC	180 (81.8)	167 (75.9)	
CC	40 (18.2)	53 (24.1)	0.16
rs7804372			
TT	131 (59.5)	116 (52.7)	
AT	71 (32.3)	86 (39.1)	
AA	18 (8.2)	18 (8.2)	0.30
TT+AT	202 (91.8)	202 (91.8)	
AA	18 (8.2)	18 (8.2)	-
rs1049337			
CC	116 (52.7)	121 (55.0)	
CT	77 (35.0)	86 (39.1)	
TT	27 (12.3)	13 (5.9)	0.06
CC+CT	193 (87.7)	207 (94.1)	
TT	27 (12.3)	13 (5.9)	0.03

X^2^ and Fisher's exact tests were performed to determine differences in genotype distribution between cases and controls.

Minor allele (T) frequency for SNP rs3779512 in the case group was 38.9%, and in the control group was 39.8% (*p*= 0.83). Genotypic distribution in cases was 40.4% (GG), 41.4% (GT) and 18.2% (TT), with F_is_= 0.13 (*p*= 0.03); in controls genotypic distribution was 37.3% (GG), 45.9% (GT) and 16.8% (TT), with F_is_=0.04 (*p*= 0.30) (Table 3).

In rs10270569, when a change C/T was reported, minor allele (T) was found in 18.4% of cases and 18.9% of controls (*p*= 0.93). Homozygotes CC, heterozygotes CT and homozygotes TT represented 67.7%, 27.7% and 4.6% in the case group, respectively; and in the control group these proportions were 64.1%, 34.1% and 1.8%. HWE was estimated in the case group (F_is_= 0.07, *p*= 0.17), and also in the control group (F_is_= -0.11, *p*= 0.97 ) (Table 3).

For SNP, rs11773845 minor allele (C) was found in 39.1% and 45.9% of cases and controls, respectively (*p*= 0.047). Among case subjects, proportions of homozygotes AA, heterozygotes AC and homozygotes CC were 40.0%, 41.8% and 18.2%, respectively. In the control group, genotypic distribution AA/AC/CC was 32.3%, 43.6% and 24.1%, in that order. In both groups F_is_ was equal to 0.12 (k= 0.04), suggesting an excess in heterozygote frequency (Table 3).

Frequency of minor allele (A) in SNP rs7804372 was 24.3% in the case group and 27.7% in the control group (*p*= 0.28). In cases, genotypic frequencies of homozygotes (TT), heterozygotes (TA) and homozygotes (AA) were 59.5%, 32.3% and 8.2%, respectively. This distribution in control subjects was 52.7%, 39.1% and 8.2%, in the same order. HWE in the case group was found with F_is_=0.12 (*p*= 0.04), and in controls with F_is_= 0.02 (*p*= 0.41) (Table 3).

Minor allele (T) in SNP rs1049337 was found in 29.8% of case subjects, and in 25.5% of controls (*p*= 0.17). Genotypic frequencies in cases were 52.7% (CC), 35.0% (CT) and 12.3% (TT); while in control subjects frequencies were 55.0% (CC), 39.1% (CT) and 5.9% (TT). Excess in heterozygotes frequency was found in cases (F_is_= 0.16, *p*= 0.01), while HWE was found in the control group (F_is_= -0.03, k= 0.71) (Table 3).

To assess differences in genotypic distribution between cases and controls, homozygotes for minor allele were compared in a recessive model. According to this approach, there were statistically significant differences between cases and controls for SNP rs1049337 (*p* <0.05) (Table 3).

## Discussion

The current research has contributed to the description of common variants in *CAV1* gene in an admixed population from Caribbean Region, which represents an initial approach to cumulate evidence on genotype/phenotype relation involving SNPs in *CAV1* and metabolic disorders that has been previously found in American-Hispanic population ^19^ ^,^ ^20^. 

In the present study, marginal differences in allele and genoptype frequencies were found, suggesting that there are little statistical significance to determine a genetic association, however further researches with a meticulous association analysis should not be discharged. According to results from this work, when case and control groups were compared in the present study, the minor allele T in rs1049337 and the homozygotes TT were found to be more frequent in cases (Table 3). In a simple way these differences between groups suggest a tendency that relates *CAV1* variations and hypertriglyceridemia in adult population, where the risk allele is acting as a recessive character. A significant Hardy-Weinberg disequilibrium was also found in the case group (F_is_= 0.16, *p*= 0.01), which indicates that an excess in heterozygotes frequency was found among these subjects, supporting findings of a recessive model that was described above. Other authors have previously analyzed this SNP in cohorts with obesity-related diseases, however a dominant model showed that there were not differences in genotype distribution between subjects with and without insulin resistance (*p*= 0.08) ^20^. Another study applied a similar design to describe association between high serum triglycerides and rs926198, nevertheless there were not differences in allele or genotype frequencies ^19^. Hence, this work brings novel evidence about this particular polymorphic variations and its behavior in groups with high serum triglycerides from an admixed population.

Since rs1049337 showed an interesting behavior, allele frequencies found in this study were compared to previous reports from reference populations in order to bring a deeper description that might be helpful in further analysis. In this regard, allele frequency for rs1049337 was similar to that reported by the HapMap project for CEU population (MAF: 27.6% vs 25.0%) (Table 4). In spite of ethnic similarities with MEX (Mexican ancestry in Los Angeles, California), it was not possible to establish a comparison with current samples from Cartagena de Indias due to the lack of data for this SNP in HapMap Project database ^35^.

**Table 4:** Allele frequencies for Single Nucleotide Polymorphisms in Caveolin 1 (*CAV1*) gene from different populations. References population were taken from HapMap project, while Cases and Controls represent sample from the present study.

SNP	Cases	Controls	CEU	MEX	YRI	HCB
rs926198						
C	0.280	0.309	0.332	0.184	0.611	0.093
T	0.720	0.691	0.668	0.816	0.389	0.907
rs3779512						
G	0.611	0.602	0.593	0.730	0.195	0.895
T	0.389	0.398	0.407	0.270	0.805	0.105
rs10270569						
C	0.816	0.811	0.726	0.780	0.732	0.965
T	0.184	0.189	0.274	0.220	0.268	0.035
rs11773845						
A	0.609	0.541	0.580	0.750	0.221	0.698
C	0.391	0.459	0.420	0.250	0.779	0.302
rs7804372						
A	0.243	0.277	0.270	0.210	0.327	0.221
T	0.757	0.723	0.730	0.790	0.673	0.779
rs1049337						
C	0.702	0.745	0.750	-	0.973	0.488
T	0.298	0.255	0.250	-	0.027	0.512

CEU: Utah residents with Northern and Western European ancestry;

MEX: Mexican ancestry in Los Angeles, California;

YRI: Yoruba in Ibadan, Nigeria;

HCB: Han Chinese in Beijing, China

In general, allelic frequencies and genotype distributions in this sample were similar to those found in groups with European ancestry living in North America (Table 4). In fact, findings from Cartagena de Indias were notably close to European frequencies even in SNPs with a high inter-population variation such as rs926198, rs3779512 and rs1049337, suggesting that Spaniard ancestry is a predominant component in contemporary population from Cartagena de Indias ^36^. 

Previous studies have described a three-hybrid admixed population in Cartagena de Indias, in which European ancestry represented more than a half of all lineages ^29^ ^,^ ^37^, thus current genotype frequencies of *CAV1* SNPs are in agreement with an elevated representation of Spanish ancestry. In this sense, it is possible that genetic association studies could be replicated employing simple fitting methods that require a conservative increase in size of study or sample size. However, following STREGA consortium recommendations ^38^, genetic stratification in Cartagena de Indias remains a concerning issue that should be included as a confounder in weighted analytical procedures. 

In spite of the historical contribution of African immigrants forced to come to the Colombian Caribbean Coast as slaves ^39^ ^-^ ^41^, this recent demographic event showed little influence on *CAV1* SNPs genetic distribution, considering that local allelic frequencies were distant to those found in reference African groups (Table 4). These results suggest that sex-biased gene flow caused by social predominance of European groups during colonial period in Latin America and the Caribbean might be responsible for some of current population genetic patterns ^42^ ^,^ ^43^.

Although results from the present study have pointed out to a relationship between *CAV1* polymorphisms and high serum triglycerides, there are some limitations that must be considered. First, this study was carried out on an admixed population where genetic stratification has been widely described, therefore a confounding effect must be discharged in further analysis ^28^ ^,^ ^37^. Second, group sizes is relatively small for complete discrimination of genetic associations, thus this study was mostly restricted to descriptive and comparative observations.

## Conclusion

Allelic and genotype frequencies described for SNPs in *CAV1* in a sample from Cartagena de Indias were similar to those observed in groups with European ancestry. Additionally, differences in allelic and genotype distributions between cases and controls found in this study settled a precedent for incoming genetic association studies with a larger sample and higher performance in analytical procedures.
