# Supplementary material for: Frequency of common polymorphisms in Caveolin 1 (CAV1 ) gene in adults with high serum triglycerides from Colombian Caribbean Coast
Source: Colomb Med (Cali). 2017 Dec 30;48(4):167–73. doi: 10.25100/cm.v48i4.2625 (PMC5896723; doi:10.25100/cm.v48i4.2625)
Supplement: Related texts

# Research article: Frecuencia de polimorfismos comunes en el gen de Caveolina 1 (***CAV1*** ) en adultos con hipertrigliceridemia en el Caribe Colombiano

## Introducción

El gen Caveolina 1 (*CAV1*) tiene una longitud de 36.4 Kb y se localiza en el cromosoma 7 (7q31.1), conformado por tres segmentos exónicos separdos por dos intrones, el cual codifica para una proteína integral de membrana de 22 KDa ^1^^-^^3^. Estos tres exones están comprendidos por 30, 165 y 342 pb, mientras que los intrones representa más del 95% de la longitud total de *CAV1* y continen más de 500 Polimorfismos de Nucleótido Simple (SNP, por las siglas en inglés para *Single Nucleotide Polymorphism*) ^1^^,^^4^. 

La proteína CAVEOLINA-1 es indispensable para la formación de la caveola en la membrana plasmática de muchos tipos celulares, sin embargo en humanos, roedores y otros animales la mayor cantidad de esta proteína se expresa en el tejido adiposo, sugiriendo que la CAVEOLINA-1 y las caveolas están involucradas en la homeostasis energética ^5^^,^^6^. Además, en estudios in vitro se ha demostrado que la expresión de CAVEOLINA-1 se incrementa significativamente durante la adipogénesis, y también se ha observado que la presencia de esta proteína es necesaria para la proliferación y diferenciación de los preadipocitos ^7^^,^^8^. 

En adipocitos, la CAVEOLINA-1 ha sido directamente relacionada directamente con el receptor de insulina como un facilitador de las fosforilación intrínseca ^9^^-^^11^, y ha sido asociada con la estabilidad del Transportador de Glucosa 4 (GLUT4) ^7^^,^^12^. Adicionalmente, la CAVEOLINA-1 y las caveolas están estrechamente implicadas en el metabolismo de triglicéridos a través de su asociación con la formación y mantenimiento de los cuerpos lipídicos, y además se sospecha que los dominios caveolares en la membrana celular son sitios de entrada para ácidos grasos y síntesis de triglicéridos ^13^^-^^15^.

Modelos animales con roedores han demostrado que la caveolina-1 es crucial en el metabolismo de triglicéridos en el tejido adiposo ya que parece estar involucrada en la distribución y dinámica de almacenamiento de los cuerpos lipídicos ^16^. En el mismo sentido, se ha encontrado que los ratones silenciados en *CAV1* al ser sometidos a una dieta alta en grasas permanecen delgados y son más propensos a desarrollar hipertrigliceridemia, así como resistencia a la insulina ^16^^,^^17^. 

En humanos, variantes comunes en *CAV1* han sido asociadas con algunos desórdenes metabólicos como resistencia a la insulina, hipertensión arterial y síndrome metabólico ^18^^-^^21^. Inicialmente dos variaciones exónicas en *CAV1* fueron asociadas con hipertensión y síndrome metabólico en una población del sur de España ^18^. Seguidamente, un SNP intrónico (rs926198) fue asociado con resistencia a la insulina y parcialmente relacionado con hipertensión en un estudio traslacional que analizó una cohorte de sujetos con ancestría Europea ^20^. Recientemente, el mismo polimorfismo fue vinculado con el síndrome metabólico, diabetes mellitus tipo 2 y bajas concentraciones de colesterol HDL en dicha cohorte con ancestría Europea, además estos hallazgos fueron replicados en un grupo Hispano genéticamente subestructurado, lo que representa la primera evidencia que relaciona a *CAV1* con alteraciones lipídicas en humanos ^19^.

A pesar del creciente cúmulo de evidencia vinculando a CAV1 con la homeostasis lipídica, aún son escasos los hallazgos que respaldan la asociación con el metabolismo de triglicéridos en grupos humanos. Los triglicéridos (o triacilglicerol), constituyen la molécula más importante para el almacenamiento de energía, se acumulan principalmente en el tejido adiposo, pero también son componentes constitutivos del plasma y suero sanguíneo ^22^. La hipertrigliceridemia es una alteración prevalente en la población adulta que generalmente está asociada a un entorno energéticamente desbalanceado ^23^. En países de medianos y bajos ingresos las dislipidemias y particularmente la hipertrigliceridemia son problemas prioritarios para la salud pública que contribuyen con la mortalidad por enfermedad cardiovascular en las poblaciones más vulnerables ^24^. 

Junto a la mayoría de los países de América Latina, Colombia ha registrado un incremento persistente en la frecuencia de hipertrigliceridemia con notables variaciones intra-nacionales que oscilan en un rango de prevalencias que va desde valores tan bajos como 19.3% hasta otros tan altos como 43.9% ^25^^,^^26^. En Cartagena de Indias, en el Caribe Colombiano, la hipertrigliceridemia fue encontrada en el 38.2% de las mujeres ^27^, y entre el 67.7 y 77.8% de la población general ^28^, lo que representa una las frecuencias más altas a nivel nacional. 

Considerando que las poblaciones del Caribe Colombiano podrían requerir estrategias novedosas para reducir el impacto de la hipertrigliceridemia sobre el bienestar colectivo, algunos han propuesto que los factores genéticos serían una fuente de datos útiles para diseñar intervenciones preventivas o abordajes terapéuticos. Sobre esta base, el presente estudio tuvo como objetivo describir las frecuencias de seis SNPs de *CAV1* en adultos con hipertrigliceridemia del Caribe Colombiano.

## Materiales y Métodos

Se llevó a cabo un estudio de casos y controles en la zona urbana de Cartagena de Indias, una población genéticamente mezclada con predominancia de ancestría Europea (60%) como consecuencia de la colonización española durante los siglos XVI al XIX ^29^. 

Fueron incluidos individuos no emparentados de ambos sexos, con 18-80 años de edad. Se excluyeron a los sujetos con alteraciones endocrinas primarias o un diagnóstico previo de enfermedad genética, desórdenes de la conducta alimentaria, hospitalización reciente, cáncer, antecedentes personales de tratamiento quirúrgico para obesidad, mujeres embarazadas y en periodo de lactancia. Todos los sujetos fueron invitados a participar voluntariamente en el estudio y se les solicitó el consentimiento informado por escrito, siguiendo las recomendaciones del Comité de Ética en Investigación de la Universidad de Cartagena. 

Los sujetos fueron seleccionados a través de un muestreo por conveniencia en los lugares de trabajo y conglomerados de viviendas. Para este procedimiento de incluyeron siete puntos de muestreo, que representan un grupo de la población con características socio-económicas mixtas y que se dedican a diversas labores (*e.g.*, administración y oficina, construcción civil, producción industrial, servicios de seguridad y docencia). Los sujetos desempleados fueron incluidos a través de un muestreo aleatorio por conglomerados que fue ejecutado en cinco áreas con características socio-demográficas también diversas.

La clasificación de los grupos de casos y controles estuvo basada en los criterios para hipertrigliceridemia de la Declaración Provisional Conjunta (JIS, por la sigla en inglés para *Joint Interim Statment*) ^30^. De acuerdo con esto, los casos fueron aquellos sujetos con concentraciones séricas de triglicéridos ≥150 mg/dL o con antecedentes clínicos de hipertrigliceridemia, mientras que los controles fueron los participantes con concentraciones inferiores a 150 mg/dL y sin diagnóstico previo de dislipidemia. 

A todos los participantes se les tomó una muestra de sangre venosa bajo condiciones de ayuno para medir las concentraciones séricas de glucosa, colesterol, triglicéridos y colesterol HDL. Los niveles de estas variables fueron determinados en el Laboratorio UNIMOL de la Universidad de Cartagena a través de ensayos colorimétricos usando protocolos estandarizados. Estos parámetros de bioquímica sanguínea fueron evaluados para brindar una mayor descripción del estado metabólico y facilitar en análisis de los resultados. Entre estas variables únicamente la trigliceridemia fue usada como criterio de discriminación entre casos y controles. 

Las variables socio-demográficas fueron registradas mediante una encuesta validada que ha sido aplicada previamente en comunidades urbanas del Caribe Colombiano ^27^^,^^28^. Los grupos de casos y controles fueron apareados por seco y edad, siguiendo los datos recolectados en esta encuesta.

Para el análisis genético, se empleó una muestra de sangre de 3 mL recolectada en tubos estériles conteniendo EDTA como anticoagulante. Estas muestras fueron refrigeradas y transportadas al Laboratorio UNIMOL en la Universidad de Cartagena donde fueron procesadas. El ADN genómico fue extraído usando un juego de reactivos comerciales (Promega Corp., Madison, USA) siguiendo los principios publicados por otros autores ^31^, y luego cuantificados por fluorometría con el Qubit 2.0 (Thermo Fisher Scientific Inc., Waltham, Estados Unidos) usando los reactivos correspondientes.

Para los ensayos de genotipificación, antes de los procedimientos de biología molecular, se seleccionaron las variantes en *CAV1* usando los datos del proyecto HapMap y el programa *Haploview 4.2* (Broad Institute, Cambridge, Estados Unidos) ^4^^,^^32^. Se emplearon los datos genómicos de la población CEU (sujetos con ancestría del Norte y Occidente de Europa residentes en Utah, Estados Unidos) como referencia para seleccionar los SNPs con una Frecuencia del Alelo Menor (MAF, por las siglas en inglés para *Minor Allele Frequency*) ≥0.25 y un coeficiente de correlación (r^2)^ ≥0.8. Según estos parámetros, seis polimorfismos fueron seleccionados para análisis posteriores (Tabla 1). 

**Tabla 1. Polimorfismos:** de Nucleótido Simple (SNP) seleccionados en el gen Caveolina 1 (CAV1) usando datos del proyecto HapMap. Se aplicaron como criterios de selección una Frecuencia de Alelo Menor (MAF) ≥0.25 y un coeficiente de correlación (r2) ≥0.8.

SNP	MAF	Function	Reference Variation
rs926198	0.332	Intron	C/T
rs3779512	0.407	Intron	G/T
rs10270569	0.274	Intron	C/T
rs11773845	0.420	Intron	A/C
rs7804372	0.270	Intron	A/T
rs1049337	0.250	3'-UTR	C/T

UTR: Región No Transcripta

Los SNPs seleccionados fueron genotipificados por Reacción en Cadena de la Polimerasa Cuantitativa, usando sondas *TaqMan* específicas (Thermo Fisher Scientific Inc., Waltham, Estados Unidos). La discriminación alélica se ejecutó automáticamente a partir de los datos finales de fluorescencia que fueron analizados con el *StepOne Real-Time PCR Software* (Thermo Fisher Scientific Inc., Waltham, Estados Unidos).

Los datos socio-demográficos, las medidas antropométricas y las concentraciones séricas fueron descritas con medidas de tendencia central y frecuencia. Según fuera apropiado, las medias fueron comparadas mediante la prueba t de Student, mientras que las frecuencias fueron comparadas con la prueba X^2^ o la prueba exacta de Fisher o de. Las frecuencias alélicas y genotípicas fueron determinadas por conteo directo, el desequilibrio de ligamientos fue estimado con el programa Arlequin 3.5 ^33^, y el Equilibrio de Hardy-Weinberg (HWE, por la sigla en inglés para *Hardy-Weinberg Equilibrium*) fue evaluado a través de los valores de F_is_ usando el programa Genetix 4.05. Las diferencias en las distribuciones genotípicas entre casos y controles fueron analizadas a través de pruebas de hipótesis nulas, como ha sido sugerido por otros autores ^34^. 

## Resultados

Se incluyó un total de 440 sujetos en el estudio (220 casos y 220 controles). Los valores promedios de edad, Índice de Masa Corporal (IMC), y de las variables antropométricas y bioquímicas se encuentran descritos en la Tabla 2. No se encontraron diferencias estadísticamente significativas en la distribución por sexo, edad y variables antropométricas entre ambos grupos. 

**Tabla 2:** Variables sociodemográficas, antropométricas y metabólicas. Los grupos fueron comparados mediante las pruebas de X^2^ o la prueba t-student, según fuera conveniente.

Variables	Casos		Controles		Valores de *p*
Sexo (hombres)	61.3%	(54.5-67.7)	66.3%	(59.6-72.5)	0.321
Edad (años)*	43.1	±12.0	42.1	±14.3	0.418
Parámetros Antropométricos*					
Índice de Masa Corporal (kg/m^2)^	26.9	±4.0	26.3	±4.4	0.139
Circunferencia abdominal (cm)	94.3	±9.5	92.4	±10.9	0.063
Perímetro de caderas (cm)	101.9	±8.5	101.9	±9.1	0.941
Presión arterial (mmHg)*					
Sistólica	114.5	±16.8	111.9	±15.1	0.082
Diastólica	76.5	±11.3	75	±10.1	0.139
Concentración séricas (mg/dL)*					
Triglicéridos	210.4	±63.4	143.2	±27.2	<0.001
Glucosa	100.7	±33.1	87.4	±25.5	<0.001
Colesterol	200.9	±68.3	178.1	±37.2	<0.001
cHDL	47.4	±16.3	42.9	±10.9	<0.001

La variable sexo fue descrita como una proporción. Intervalo de Confianza del 95%.

* Promedio ± Desviación estándar

En el SNP rs926198, el alelo menor (C) fue encontrado en el 27.9% de los casos y en el 30.9% de los controles (*p*= 0.34). En el grupo de casos, los genotipos TT, TC y CC se encontraron en el 52.7%, 38.6% y 8.6% de los sujetos respectivamente; mientras que en el grupo de controles estos genotipos se encontraron en el 50.0%, 38.2% and 11.8%, respectivamente. Ambos grupos se encontraban en equilibrio de Hardy-Weinberg con valores de F_is_ de 0.04 (*p*= 0.31) y 0.10 (*p*= 0.07) para los casos y los controles respectivamente (Tabla 3).

**Tabla 3:** Distribución genotípica de los SNPs en el gen de Caveolina 1 (*CAV1*) en sujetos con hipertrigliceridemia (casos) y controles. Se aplicó un modelo recesivo para la comparación de las diferencias entre ambos grupos (casos y controles).

Distribución genotípica	Cases	Controles	Valores de *p*
rs926198			
TT	116 (52.7)	110 (50.0)	
TC	85 (38.6)	84 (38.2)	
CC	19 (8.6)	26 (11.8)	0.53
TT+TC	201 (91.4)	194 (88.2)	
CC	19 (8.6)	26 (11.8)	0.34
rs3779512			
GG	89 (40.4)	82 (37.3)	
GT	91 (41.4)	101 (45.9)	
TT	40 (18.2)	37 (16.8)	0.62
GG+GT	180 (81.8)	183 (83.2)	
TT	40 (18.2)	37 (16.8)	0.70
rs10270569			
CC	149 (67.7)	141 (64.1)	
CT	61 (27.7)	75 (34.1)	
TT	10 (4.6)	4 (1.8)	0.12
CC+CT	210 (95.4)	216 (98.2)	
TT	10 (4.6)	4 (1.8)	0.17
rs11773845			
AA	88 (40.0)	71 (32.3)	
AC	92 (41.8)	96 (43.6)	
CC	40 (18.2)	53 (24.1)	0.15
AA+AC	180 (81.8)	167 (75.9)	
CC	40 (18.2)	53 (24.1)	0.16
rs7804372			
TT	131 (59.5)	116 (52.7)	
AT	71 (32.3)	86 (39.1)	
AA	18 (8.2)	18 (8.2)	0.30
TT+AT	202 (91.8)	202 (91.8)	
AA	18 (8.2)	18 (8.2)	-
rs1049337			
CC	116 (52.7)	121 (55.0)	
CT	77 (35.0)	86 (39.1)	
TT	27 (12.3)	13 (5.9)	0.06
CC+CT	193 (87.7)	207 (94.1)	
TT	27 (12.3)	13 (5.9)	0.03

Para determinar las diferencias de la distribución genotípica entre el grupo de casos y de controles se aplicó la prueba de X^2^ y la prueba exacta de Fisher.

La frecuencia del alelo menor (T) para el SNP rs3779512 en el grupo de casos fue de 38.9%, y de 39.8% en el de control (*p*= 0.83). La distribución genotípica en el grupo de casos fue de 40.4% (GG), 41.4% (GT) y 18.2% (TT), con F_is_= 0.13 (*p*= 0.03); en los controles, la distribución fue de 37.3% (GG), 45.9% (GT) y 16.8% (TT), con F_is_=0.04 (*p*= 0.30) (Tabla 3).

En rs10270569, donde ocurre el cambio C/T, la frecuencia del alelo menor (T) fue de 18.4% en los casos y del 18.9% en los controles (*p*= 0.93). Los homocigotos CC, heterocigotos CT y homocigotos TT representaron el 67.7%, 27.7% y 4.6% en el grupo de casos, respectivamente; y en el grupo de control estas proporciones fueron 64.1%, 34.1% y 1.8% respectivamente. Se estimó el HWE en el grupo de casos (F_is_= 0.07, *p*= 0.17), y también en el de controles (F_is_= -0.11, *p*= 0.97 ) (Tabla 3).

En el SNP rs11773845 el alelo menor (C) fue encontrado en el 39.1% y 45.9% de los casos y controles, respectivamente (*p*= 0.047). Entre los casos, las proporciones de homocigotos AA, heterocigotos AC y homocigotos CC fueron de 40.0%, 41.8% y 18.2%, respectivamente. En el grupo de controles, la distribución genotípica de AA/AC/CC fue de 32.3%, 43.6% and 24.1%, en ese orden. En ambos grupos, el valor de F_is_ fue igual a 0.12 (*p*= 0.04), sugiriendo un exceso en la frecuencia de heterocigotos (Tabla 3).

La frecuencia del alelo menor (A) en el SNP rs7804372 fue de 24.3% en el grupo de casos y de 27.7% en el grupo de controles (*p*= 0.28). En los casos, las frecuencias genotípicas de los homocigotos (TT), heterocigotos (TA) y homocigotos AA fueron 59.5%, 32.3% y 8.2%, respectivamente. Esta distribución en el grupo de controles fue de 52.7%, 39.1% y 8.2%, en el mismo orden. El grupo de casos se encontró en HWE con un F_is_= 0.12 (*p*= 0.04), y en los controles con un F_is_= 0.02 (*p*= 0.41) (Tabla 3).

El alelo menor (T) en el SNP rs1049337 fue encontrado en un 29.8% de los casos, y en un 25.5% de los controles (*p*= 0.17). En los casos, las frecuencias genotípicas fueron de 52.7% (CC), 35.0% (CT) y 12.3% (TT); mientras que en los controles las frecuencias se encontraron en 55.0% (CC), 39.1% (CT) y 5.9% (TT). Se encontró un exceso en la frecuencia de heterocigotos en el grupos de casos (F_is_= 0.16, *p*= 0.01), mientras que en el grupo control se encontró en HWE (F_is_= -0.03, *p*= 0.71) (Tabla 3).

Para evaluar las diferencias en la distribución genotípicas entre los casos y controles, se compararon los homocigotos para el alelo menor mediante un modelo recesivo. De acuerdo con este abordaje, se encontraron diferencias estadísticamente significativas entre los grupos de casos y controles para el SNP rs1049337 (*p* <0.05) (Tabla 3).

## Discusión

La presente investigación contribuyó con las descripción de variantes comunes en el gen *CAV1* en una población genéticamente estratificada de la región caribe, lo cual representa un enfoque inicial para acumular evidencia sobre la relación genotipo/fenotipo entre SNPs en *CAV1* y trastornos metabólicos, que han sido previamente encontrados en población hispanoamericana ^19^^,^^20^. 

En el presente estudio, se encontraron diferencias marginales en las frecuencias alélicas y genotípicas, lo que sugiere que hay poca significancia estadística para determinar una asociación genética, sin embargo, no se debe descartar la realización de más investigaciones que permitan esclarecer este vínculo a través de análisis de asociación más profundos. De acuerdo con los resultados de este trabajo, tras realizar la comparación entre los grupos de casos y controles, se observó que el alelo menor T en rs1049337 y los homocigotos TT fueron más frecuentes en el grupo de casos (Tabla 3). Estas diferencias entre los grupos sugieren una tendencia que relaciona variaciones en *CAV1* con hipertrigliceridemia en la población adulta, encontrándose el alelo de riesgo en su forma recesiva. En el grupo de casos se encontró un significativo desequilibrio de Hardy-Weinberg (F_is_= 0.16, *p*= 0.01), lo que indica un exceso en la frecuencia de heterocigotos entre estos sujetos, apoyando los hallazgos del modelo recesivo descrito arriba. Otros autores han analizado previamente este SNP en cohortes con enfermedades relacionadas con la obesidad, sin embargo, un modelo dominante mostró que no hubo diferencias en la distribución del genotipo entre sujetos con y sin resistencia a la insulina (*p*= 0.08) ^20^. Otro estudio aplicó un diseño similar para describir la asociación entre hipertrigliceridemia y rs926198, sin embargo, no hubo diferencias en las frecuencias alélicas o genotípicas ^19^. Por lo tanto, este trabajo aporta evidencia novedosa acerca de estas particulares variaciones polimórficas y su comportamiento en grupos con hipertrigliceridemia en una población genéticamente estratificada.

Teniendo en cuenta el interesante comportamiento mostrado por rs1049337, las frecuencias alélicas encontradas en este estudio fueron comparadas con los informes anteriores de las poblaciones de referencia, con el fin de brindar una descripción más profunda que pudiera ser útil en análisis posteriores. En este sentido, la frecuencia de los alelos para rs1049337 fue similar a la reportada por el proyecto *HapMap* para la población de CEU (MAF: 27.6% vs 25.0%) (Tabla 4). A pesar de las similitudes étnicas con MEX (ascendencia mexicana en Los Angeles, California), no fue posible establecer una comparación con muestras actuales de Cartagena de Indias debido a la falta de datos para este SNP en la base de datos del Proyecto HapMap ^35^.

**Tabla 4:** Frecuencias alélicas paras los SNPs en el gen de Caveolina 1 (*CAV1*) gene de diferentes poblaciones. La población referencia fue tomada del Proyecto *HapMap*, mientras que los casos y controles representan la muestra del presente estudio.

SNP	Casos	Controles	CEU	MEX	YRI	HCB
rs926198						
C	0.280	0.309	0.332	0.184	0.611	0.093
T	0.720	0.691	0.668	0.816	0.389	0.907
rs3779512						
G	0.611	0.602	0.593	0.730	0.195	0.895
T	0.389	0.398	0.407	0.270	0.805	0.105
rs10270569						
C	0.816	0.811	0.726	0.780	0.732	0.965
T	0.184	0.189	0.274	0.220	0.268	0.035
rs11773845						
A	0.609	0.541	0.580	0.750	0.221	0.698
C	0.391	0.459	0.420	0.250	0.779	0.302
rs7804372						
A	0.243	0.277	0.270	0.210	0.327	0.221
T	0.757	0.723	0.730	0.790	0.673	0.779
rs1049337						
C	0.702	0.745	0.750	-	0.973	0.488
T	0.298	0.255	0.250	-	0.027	0.512
			

CEU: residentes de Utah con ascendencia del Norte y Occidente de Europa;

MEX: ascendencia mexicana en Los Ángeles, California;

YRI: Yoruba en Ibadan, Nigeria;

HCB: Chinos Han en Beijing, China.

En general, las frecuencias alélicas y las distribuciones de genotipos en esta muestra fueron similares a las encontradas en los grupos con ancestría europea que viven en América del Norte (Tabla 4). De hecho, los hallazgos encontrados en Cartagena de Indias fueron notablemente cercanos a las frecuencias Europeas incluso en SNPs con una alta variación interpoblacional como en el caso de los SNPs rs926198, rs3779512 y rs1049337, lo que sugiere que la ascendencia española es un componente predominante en la población contemporánea de Cartagena de Indias ^36^. 

Estudios previos han descrito una población mixta compuesta tres subpoblaciones en Cartagena de Indias, donde la ascendencia Europea representa más de la mitad de todos los linajes ^29^^,^^37^, por lo que las frecuencias genotípica actuales de los SNP en *CAV1* se encuentran acorde con la elevada representación de la ascendencia española. En este sentido, es posible que los estudios de asociación genética puedan ser replicados empleando métodos de ajuste simples que requieran un aumento conservador en el poder del estudio o en el tamaño de la muestra. Sin embargo, siguiendo las recomendaciones del consorcio STREGA ^38^, la estratificación genética en Cartagena de Indias sigue siendo una cuestión a tener en cuenta que debe ser incluida como un factor de confusión en la ponderación de los procedimientos analíticos.

A pesar de la contribución histórica por parte de los inmigrantes Africanos que fueron transportados forzosamente como esclavos al Caribe Colombiano ^39^^-^^41^, este reciente evento demográfico mostró poca influencia en la distribución genética de los SNPs en C*AV1*, considerando que las frecuencias alélicas locales se encontraron distantes a las observadas en grupos Africanos de referencia (Tabla 4). Estos resultados sugieren que el flujo genético influenciado por el sexo causado por el predominio social de los grupos Europeos durante el período colonial en América Latina y el Caribe podría ser responsable de algunos de los patrones genéticos actuales de la población ^42^^,^^43^.

Aunque los resultados del presente estudio han señalado una relación entre los polimorfismos en *CAV1* e hipertrigliceridemia, hay algunas limitaciones que deben ser consideradas. En primer lugar, este estudio se llevó a cabo en una población multiétnica, donde la estratificación genética ha sido ampliamente descrita, por lo tanto, el efecto de confundidor debe ser tenido en cuenta análisis posteriores ^29^^,^^37^. En segundo lugar, el tamaño de los grupos es relativamente pequeño para una completa discriminación de las asociaciones genéticas, por lo que este estudio se limitó principalmente a observaciones descriptivas y comparativas.

## Conclusión

Las frecuencias alélicas y genotípicas descritas para los SNPs en *CAV1* en una muestra de Cartagena de Indias fueron similares a las observadas en grupos con ascendencia europea. Adicionalmente, las diferencias en las distribuciones alélicas y de genotipos entre los casos y los controles encontradas en este estudio, establecen un precedente para próximos estudios de asociación genética con una más amplia muestra y un más alto rendimiento en los procedimientos analíticos.
